# Recovery of Strength After Exercise-Induced Muscle Damage in Vegetarians Consuming the Upper and Lower Ends of Protein Recommendations for Athletes

**DOI:** 10.3390/nu17061046

**Published:** 2025-03-17

**Authors:** Nicole Presti, Todd C. Rideout, Jennifer L. Temple, Brian Bratta, David Hostler

**Affiliations:** 1 Center for Research and Education in Special Environments, Exercise and Nutrition Sciences Department, University at Buffalo, 4 Sherman Annex, Buffalo, NY 14214, USA; npresti@buffalo.edu; 2 Exercise and Nutrition Sciences Department, University at Buffalo, G10 Farber Hall, Buffalo, NY 14214, USA; rideout@buffalo.edu; 3 Exercise and Nutrition Sciences Department, University at Buffalo, 114 Sherman Hall, Buffalo, NY 14214, USA; jltemple@buffalo.edu; 4 Athletics Department, University at Buffalo, 102 Alumni Arena, Buffalo, NY 14260, USA; bbratta@buffalo.edu

**Keywords:** plant-based, Lacto-ovo, EIMD, delayed onset muscle soreness, DOMS

## Abstract

**Background/Objective**: Plant-based protein is less bioavailable than animal protein. It is unclear if the protein recommendations for athletes should be increased when following a vegetarian diet. This study’s purpose is to document the recovery of strength and power, as well as to assess soreness after exercise-induced muscle damage (EIMD), in people following a vegetarian diet while consuming the lower (1.2 g/kg/day) and upper (2.0 g/kg/day) ends of protein recommendations for athletes. **Methods**: In this crossover design study, subjects were randomly assigned to consume 1.2 or 2.0 g/kg/day of protein and were supplemented up to their allotted amount with pea protein. Sixteen male (*n* = 9) and female (*n* = 7) subjects (24 ± 2 yr, 170 ± 7 cm, 68.2 ± 10.0 kg) performed a single-leg vertical jump and maximal isometric and isokinetic knee extension prior to, and five days following, EIMD. The quadricep muscle was injured by completing 10 × 10 eccentric contractions on an isokinetic dynamometer. The opposite condition was performed after a 2-week washout period. **Results**: There was a difference over time (*p* < 0.001), but not between conditions, for isometric strength (*p* = 0.92), vertical jump (*p* = 0.78), concentric strength at 60 (*p* = 0.92), 180 (*p* = 0.91), and 240 degrees per second (*p* = 0.90). There was a difference over time (*p* < 0.001), but not between groups, for pressure pain threshold while sitting (*p* = 0.74) and standing (*p* = 0.94), and the 10 cm visual analog scale completed while walking (*p* = 0.10), sitting (*p* = 0.32), and standing (*p* = 0.15). **Conclusions**: There was no difference in recovery of strength, power, and soreness after EIMD in people who follow a vegetarian diet while consuming the lower and upper ends of protein recommendations for athletes.

## 1. Introduction

Exercise-induced muscle damage (EIMD) occurs after an unaccustomed exercise or exertion. Signs and symptoms of EIMD include muscle soreness, stiffness, tenderness, pain, and swelling [1,2]. There is decreased muscle function, which impairs strength, power, speed, and range of motion [2]. EIMD occurs immediately after exercise, but the side effects can last for several days [3,4] and coincide with delayed onset muscle soreness (DOMS). DOMS typically appears 12–24 h after exercise, peaks between 24 and 72 h, and resolves in 5–7 days [5,6]. EIMD and DOMS can decrease performance if insufficient time is available for recovery. Elite athletes typically perform 15–30 h of training per week, and team sports may play games every 2–3 days [7].

Proper nutrition can be employed to help athletes recover more quickly from exercise [8,9]. The dietary reference intake (DRI) in the United States is a set of recommended reference values for nutrients for the general population. The DRI can be adjusted to meet the needs of the individual. For example, the DRI for iron is 1.8 times higher for people following a plant-based diet due to non-heme iron being less bioavailable than heme iron [10]. Similarly, the bioavailability of plant-based protein is lower when compared to animal protein; however, the protein recommendations for people following a plant-based diet and people who consume meat are the same [11].

Kniskern et al. investigated protein digestibility in vegetarian diets to determine if the DRI for protein in vegetarians should be increased. Twenty-two women completed a four-day diet recall. The protein digestibility of their diets was calculated by multiplying the DRI reference value to the protein percentage for that group (cereals, legumes, nuts/seeds, fruits/vegetables, and dairy/egg). Their study concluded vegetarians who consumed a low amount of animal protein would need to consume extra protein to meet the recommendation. In application, if a woman’s recommended intake for protein was 0.8 g/kg/day, she would need to consume 1.0 g/kg/day of protein to meet the recommended intake if consuming a vegetarian diet. This equates to about an extra 12 g of protein per day in a 60 kg individual [12].

While the DRI for protein in the general population is 0.8 g/kg/day, an athlete’s protein needs are higher, ranging from 1.2 to 2.0 g/kg/day [13]. Ciuris et al. investigated overall protein absorbability using the Digestible Indispensable Amino Acid Score (DIAAS) in vegetarian and omnivorous endurance athletes by having them complete a 7-day diet recall. In this study, the omnivore group had higher protein absorbability with an 11% higher DIAAS, as well as more available protein per gram (43% more) and grams/kilogram (27% more) when compared to the vegetarian group. In application, a 64 kg vegetarian athlete would need to consume an extra 10 g of protein per day to meet the 1.2 g/kg/day recommendation and an extra 22 g of protein per day to meet the 1.4 g/kg/day recommendation [14].

The purpose of this study is to determine the impact of consuming the lower and upper ends of the protein recommendations for athletes on the recovery of strength, power, and soreness after EIMD in people following a vegetarian diet. We hypothesized that vegetarians consuming 2.0 g/kg/day of protein would recover faster when compared to consuming 1.2 g/kg/day, which would suggest that the DRI for protein in vegetarian athletes may need to be increased.

## 2. Materials and Methods

Using a crossover design, subjects completed 18 study visits split into two phases. Phase one occurred during visits 1–11, and phase two during visits 12–18. There was a two-week washout period between phases. Subjects were randomly assigned to consume either 1.2 or 2.0 g/kg/day of protein during phase one and the opposite amount during phase two. During both phases, habitual protein intake was determined by analyzing a 3-day diet recall, and subjects were supplemented up to their assigned amount using a pea protein isolate. A muscle damage protocol was completed, and recovery was recorded for the following 5 days. All study procedures were approved by the University at Buffalo Institutional Review Board.

### 2.1. Subjects

The power calculation was created by G*Power 3.1.9.7 based on previous studies measuring muscle damage using isokinetic dynamometry. Six subjects were sufficient to identify differences in the primary outcomes (a = 0.05, b = 0.90). Sixteen subjects (7 females, 9 males) completed the study and were used in the analysis. Subjects were included if they were aged 18–39 and had no neuromuscular disease or lower leg injuries in the previous year. Subjects could not use tobacco or be pregnant, lactating, trying to become pregnant, or have any contraindications to exercise. They must have been following a plant-based diet for a minimum of three months. Subjects could consume eggs and dairy but had to exclude meat, poultry, pork, and seafood. Subject demographics are displayed in Table 1.

### 2.2. Visit 1: Diet Recall

After providing informed consent, subjects were instructed by a registered dietitian on how to complete the 3-day diet recall consisting of one weekday, one weekend day, and one additional day. Subjects were provided a small kitchen scale to assist with measuring food. The diet recalls were analyzed with Nutrition Data System for Research (NDSR) (version 2023 © 2023 Regents of the University of Minnesota, Minneapolis, MN, USA). The diet recalls were used to determine baseline protein intake and the required protein supplementation. The first diet recall was conducted before the start of the muscle damage protocol in phase one. The second diet recall was conducted during the two-week washout period.

### 2.3. Visits 2–5: Start of Phase One/Baseline Measurements/Familiarization

Height, mass, calculated BMI, resting heart rate, and blood pressure were recorded during visit 2. Baseline measurements (described below) were repeated on visits 2–5 to familiarize subjects with the procedures and to ensure maximal effort was given on each test.

### 2.4. Power

Jump height was measured by performing a single-leg vertical jump on a Just Jump System (Probotics, Inc., Huntsville, AL, USA). Subjects stood on the jump mat on their dominant leg. Subjects placed their hands on their hips and jumped vertically for a maximal effort. The highest jump out of three attempts was recorded.

### 2.5. Isometric Contractions

Subjects performed a knee extensor force test on a Biodex Medical Systems, Rev 4.63 10 May 2018, Shirley, NY, USA (Biodex dynamometer). Tests were conducted by a single researcher whose coefficient of variation was previously determined to be under 5%. Subjects completed three maximal isometric contractions of the knee extensor (at 90 degrees knee flexion) on their dominant leg for six seconds. There was a 2 min rest between contractions. The highest torque was recorded.

### 2.6. Concentric Contractions at 60, 180, and 240 Degrees per Second

Subjects performed a concentric torque test on the Biodex Dynamometer. The Biodex measured knee extensor torque produced by the dominate leg by immobilizing the thigh and the torso. Subjects completed five maximal concentric contractions of the knee flexor and extensor at 60, 180, and 240 degrees per second with five seconds between each contraction. The highest torque of three attempts was recorded.

### 2.7. Supplements

Subjects were randomly assigned to consume the lower (LP) (1.2 g/kg/day) and higher (HP) (2.0 g/kg/day) ends of protein recommendations for athletes. Baseline protein intake was previously determined from the 3-day diet recall, and subjects were supplemented to their allotted amount with unflavored pea protein isolate. Pea protein was chosen over soy because soy is a major allergen. During visit 6, subjects were provided with a shaker bottle and six bags containing their daily pre-measured protein supplement. Daily protein supplement was consumed from visit 6 to visit 11. They had the entire day to finish their supplement and were instructed to divide their daily allowance of protein into more than one shake if they required more than 30 g of daily protein supplement. Subjects were not restricted to what other non-protein ingredients were placed in the shake.

### 2.8. Visit 6: Supplement/Muscle Damage Protocol

The muscle damage protocol consisted of a series of muscle contractions on the isokinetic dynamometer. Subjects performed 10 sets of 10 eccentric contractions with 30 s of rest between each set. After the muscle damage protocol, baseline measurements were repeated, and soreness was assessed using a 10 cm visual analog scale (VAS) and by performing a pain upon use test.

### 2.9. 10 cm Visual Analog Scale

Subjects were instructed to make a mark on a 10 cm line expressing how the dominant quadricep felt while sitting, standing, and walking. The left end of the line represented no pain, while the right end of the line represented the worse pain possible.

### 2.10. Pain Upon Use Test

A pressure algometer was used to record the pressure pain threshold (PPT) when sitting and standing. The pressure algometer applied gradual pressure into the dominant quadricep. Subjects were instructed to tell their study team leader when pressure became pain by saying “now”. Pressure was withdrawn and recorded.

### 2.11. Visits: 7–11

Once the muscle damage protocol was completed, there were five consecutive days of post testing which were scheduled 22–24 h apart from each other. During each of these visits, subjects repeated baseline measurements, and soreness were assessed.

### 2.12. Washout Period

The washout period lasted two weeks. During the washout period, subjects were instructed to not make any deviations from their normal eating habits. Subjects completed a second 3-day diet recall to confirm baseline protein intake. Three days before the start of phase two, subjects were instructed to refrain from performing resistance training exercise.

### 2.13. Visit 12: Start of Phase Two/Reestablishing Baseline

A pregnancy test was conducted for female subjects, and baseline measurements were reestablished.

### 2.14. Visit 13

The muscle damage protocol, baseline measurements, and soreness assessments were repeated. Subjects received their daily pre-measured protein supplement consuming the opposite amount that they were assigned to during phase 1. Subjects started consuming the supplement during visit 13 and ended on visit 18.

### 2.15. Visit 14–18

Baseline measures were repeated to measure recovery.

### 2.16. Statistical Analysis

Two-way ANOVA was used to analyze study outcomes. Due to a main effect of time, a post-hoc paired *t*-test was performed for each diet. To analyze diet, a *t*-test was performed. Normality was confirmed prior to performing the analysis, and the data were checked for outliers. A mixed-effect analysis was run on the pressure algometer data due to one subject missing one data point. Statistical software GraphPad Prism 10 and Excel were used to analyze data. Significance was set at *p* < 0.05.

## 3. Results

### 3.1. Strength: Isometric

**Figure 1:**
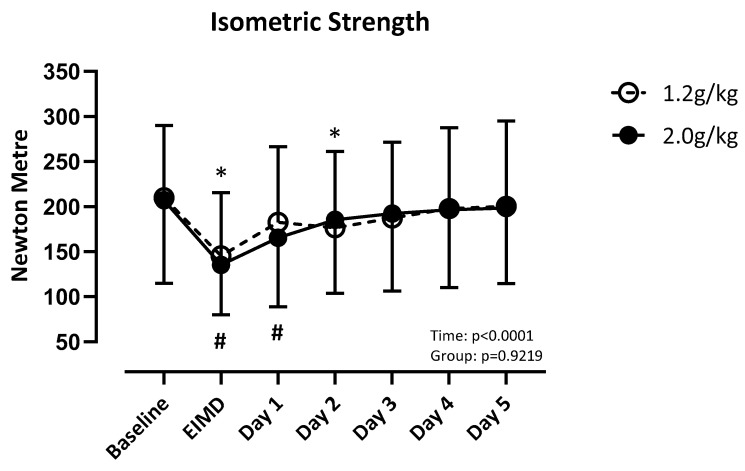
Absolute values of quadricep isometric strength compared to baseline. Significance set at *p* < 0.05. EIMD = day of the exercise-induced muscle damage protocol. * = different from baseline when consuming 1.2 g/kg. # = different from baseline when consuming 2.0 g/kg.

There was a difference over time (F (3.445, 103.3) = 30.32; *p* < 0.001), but not between groups (F (1, 30) = 0.010; *p* = 0.92), for quadricep isometric strength. LP recovered by post day 3, and HP recovered by post day 2 (Figure 1).

### 3.2. Power: Vertical Jump

**Figure 2:**
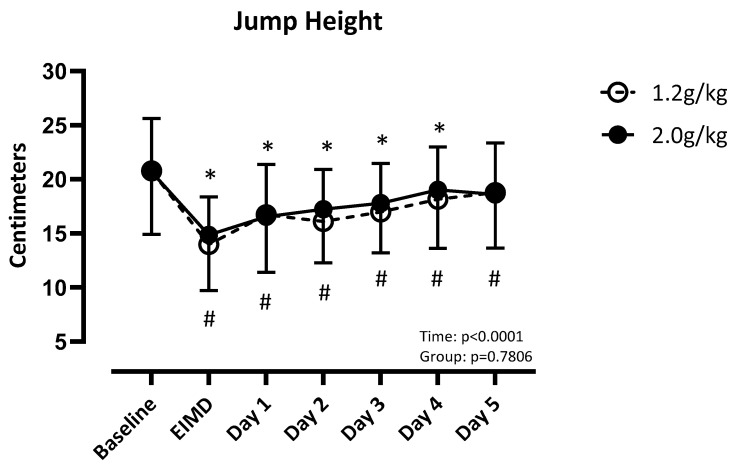
Absolute values of jump height compared to baseline. Significance set at *p* < 0.05. EIMD = day of the exercise-induced muscle damage protocol. * = different from baseline when consuming 1.2 g/kg. # = different from baseline when consuming 2.0 g/kg.

There was a difference over time (F (2.98, 89.42) = 40.58; *p* < 0.001), but not between groups (F (2.981, 89.42) = 40.58; *p* = 0.78), for the single-leg vertical jump. LP recovered by post day 5, but the HP did not recover by post day 5 (Figure 2).

### 3.3. Concentric Strength

**Figure 3:**
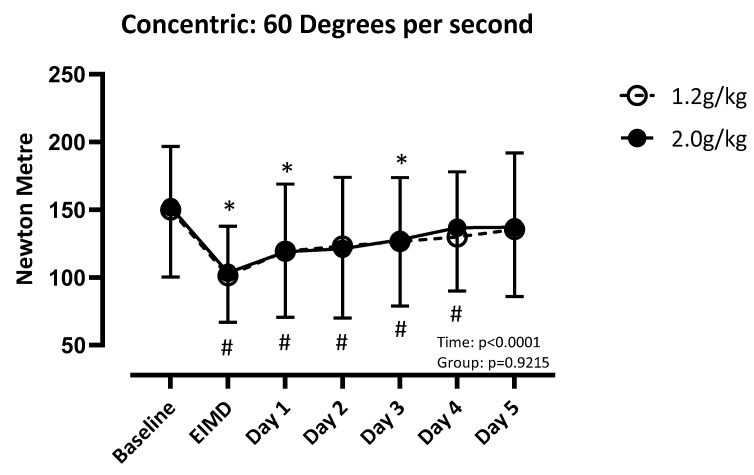
Absolute values of quadricep concentric strength at 60 degrees per second compared to baseline. Significance set at *p* < 0.05. EIMD = day of the exercise-induced muscle damage protocol. * = different from baseline when consuming 1.2 g/kg. # = different from baseline when consuming 2.0 g/kg.

**Figure 4:**
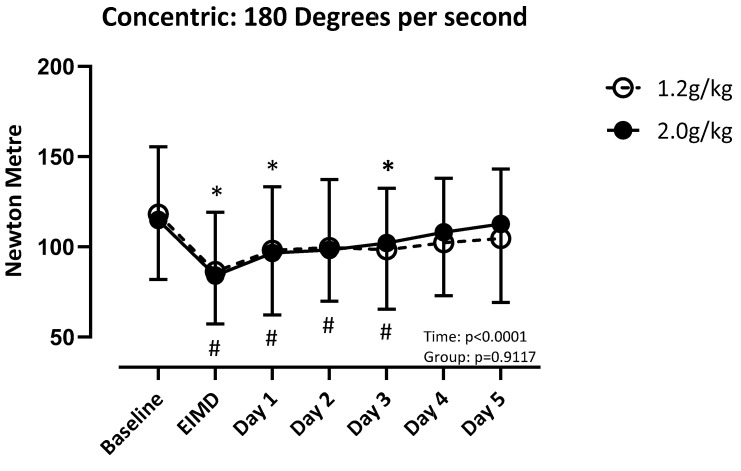
Absolute values of quadricep concentric strength at 180 degrees per second compared to baseline. Significance set at *p* < 0.05. EIMD = day of the exercise-induced muscle damage protocol. * = different from baseline when consuming 1.2 g/kg. # = different from baseline when consuming 2.0 g/kg.

**Figure 5:**
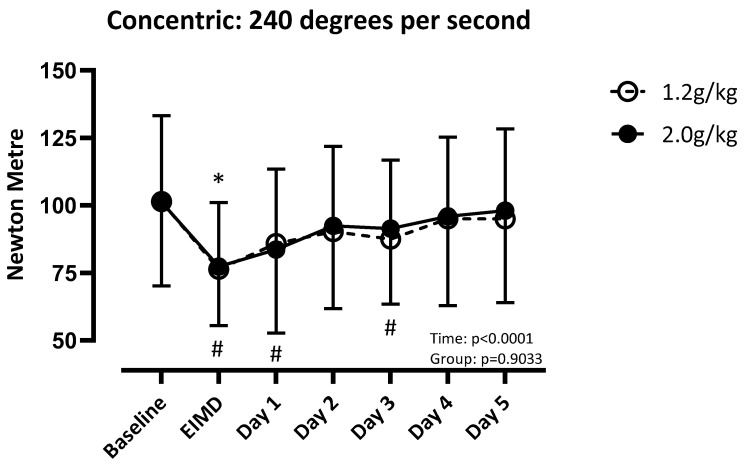
Absolute values of quadricep concentric strength at 240 degrees per second compared to baseline. Significance set at *p* < 0.05. EIMD = day of the exercise-induced muscle damage protocol. * = different from baseline when consuming 1.2 g/kg. # = different from baseline when consuming 2.0 g/kg.

There was a difference over time (F (3.756, 112.7) = 31.51; *p* < 0.001), but not between groups (F (1, 30) = 0.01; *p* = 0.92), for quadriceps concentric strength at 60 degrees per second. LP recovered by post day 4, and HP recovered by post day 5 (Figure 3). There was a difference over time (F (3.932, 118.0) = 22.03; *p* < 0.001), but not between groups (F (1, 30) = 0.013; *p* = 0.91), for concentric strength of the quadricep at 180 degrees per second. Both groups recovered by post day 4 (Figure 4). There was a difference over time (F (4.081, 122.4) = 20.41; *p* < 0.001), but not between groups (F (1, 30) = 0.02; *p* = 0.90), for concentric strength at 240 degrees per second. LP recovered by post day 1, and HP recovered by post day 4 (Figure 5).

### 3.4. Soreness

There was a difference over time (*p* < 0.001), but not between groups, when investigating the pressure pain threshold while sitting (F (1, 30) = 0.11; *p* = 0.74) and standing (F (1, 30) = 0.006; *p* = 0.94). For sitting, LP differed from EIMD on post day 4, while HP differed from EIMD on post day 2. For standing, LP differed from EIMD on post day 4, while HP did not differ from EIMD. There was a difference over time (*p* < 0.001), but not between groups, when evaluating the VAS for walking (F (1, 30) = 2.95; *p* = 0.10), sitting (F (1, 30) = 1.03; *p* = 0.32), and standing (F (1, 30) = 2.2; *p* = 0.15). For walking, both groups differed from EIMD on post day 4. For sitting and standing, LP differed from the EIMD on post day 4, and HP differed on post day 5.

### 3.5. Diet

Habitual intake of calories (*p* = 0.84), protein (*p* = 0.44), animal protein (*p* = 0.94), plant protein (*p* = 0.29), carbohydrates (*p* = 0.65), and fat (*p* = 0.63) did not change between phases (Table 2). Essential amino acids (*p* > 0.05) were not different between phases (Table 3).

## 4. Discussion

In this study, the recovery of isometric strength, concentric strength, power, and soreness did not differ among vegetarian subjects when consuming 1.2 or 2.0 g/kg/day of protein. Our results suggest that the DRI for protein intake in athletes does not need to be increased for athletes following a vegetarian diet. Although we cannot comment outside the context of recovery from EIMD, our results contradict Kniskern et al. and Ciuris et al., who concluded that vegetarians need to consume more protein based on an analysis of protein bioavailability in vegetarian diets [12,14].

A possible reason for the conflicting results is from the different study design. Kniskern et al. and Ciuris et al. drew their conclusions by investigating the bioavailability of protein in vegetarian diets, while our study used physical assessments [12,14]. Another possibility is that the pea protein supplement may have increased the bioavailability of protein in the diet and may not represent the bioavailability of protein in a typical vegetarian diet. Anti-nutritional factors found in plants reduce the maximum utilization of protein. While peas contain anti-nutritional factors such as tannins, which reduce protein absorption [15,16], these anti-nutritional factors are removed when producing a pea protein supplement, which increases bioavailability [17]. Another consideration is the subject pool in each study; Kniskern et al. recruited only females, while this study and Ciuris et al.’s study recruited males and females [12,14]. Ciuris et al. recruited competitive endurance runners, while this study and Kniskern et al. did not recruit trained subjects [12,14]. These differences could be an additional explanation for the conflicting results [12,14]. In the present report, subjects were supplemented up to the target of 1.2 or 2.0 g/kg/day of protein using a pea protein isolate. During the LP phase, two subjects were already consuming 1.2 g/kg/day of protein. Fourteen subjects in the 1.2 g/kg/day condition and all subjects in the 2.0 g/kg/day condition were supplemented with pea protein, which may have increased the overall bioavailability of protein in their diet, which improved recovery.

A limitation to this study is the lack of a control group. Our results showed that the lower end of protein recommendations for athletes was sufficient for muscle recovery in people following a vegetarian diet. However, vegetarians could recover from EIMD at a slower rate due to the underconsumption of protein. As shown in Table 2, the average habitual protein intake of our subjects was 0.9 g/kg/day which is less than the minimum recommended amount of 1.2 g/kg/day. This could have been investigated with a control group, but the focus of this investigation was the recommendations for athletes. A second limitation is that we could not confirm that the subjects consumed all the protein shakes or that their habitual protein intake was truly reflected in the diet recall. Subjects were given pre-measured bags of protein and were instructed to consume the protein by the end of the day, but they did not have to consume the protein in front of a member of the study team. Monitoring intake would have required subjects being admitted to a clinical research center, which was not possible. The subjects were closely coached by a registered dietitian and did provide two 3-day diet recalls, which reported similar results.

## 5. Conclusions

There was no difference in the recovery of strength, power, and soreness after EIMD in people following a vegetarian diet while consuming the lower and upper ends of protein recommendations for athletes. Our results would suggest that vegetarian athletes do not need to consume extra protein to compensate for the lower bioavailability of plant protein if consuming at least the lower end of protein recommendation (1.2 g/kg/day) for athletes.

## 6. Future Recommendations

Future research should investigate the impact following a vegetarian diet has on muscle recovery without the use of supplementation or creating a diet where protein combinations are ideal. In addition, future research should investigate the impact a vegan diet has on muscle recovery. While we chose to recruit vegetarian subjects to match the subject pool in the studies by Kniskern et al. and Ciuris et al., we cannot generalize out findings to vegan athletes [12,14].

## Figures and Tables

**Table 1:** Subject demographics.

Anthropometrics	
Height	170 ± 6.8 cm
Mass	68.2 ± 10 kg
BMI	23.5 ± 2.5 kg/m^2^
Age	23.5 ± 2.4 yr
Sex	
Female	*n* = 7
Male	*n* = 9
Race	
Asian	*n* = 9
White	*n* = 5
Black	*n* = 1
Mix	*n* = 1

**Table 2:** Diet analysis.

Diet (g)	Average	1.2 g/kg	2.0 g/kg
Energy	1833.2 ± 523.5	1824.6 ± 483.3	1841.8 ± 576.8
Fat	65.0 ± 29.9	63.0 ± 25.9	67.0 ± 34.2
Carbohydrates	255.0 ± 85.5	258.3 ± 88.5	251.7 ± 85.1
Protein	62.4 ± 20.3	60.8 ± 16.1	64.0 ± 24.3
Animal protein	19.5 ± 13.8	19.4 ± 11.8	19.5 ± 16
Plant protein	42.9 ± 16.4	41.4 ± 15.5	44.5 ± 17.7
Diet (g/kg)	Average	1.2 g/kg	2.0 g/kg
Energy	27.2 ± 7.8	27.2 ± 7.8	27.2 ± 8.2
Fat	1.1 ± 0.5	0.9 ± 0.4	1.2 ± 0.6
Carbohydrates	3.8 ± 1.3	3.9 ± 1.4	3.7 ± 1.3
Protein	0.9 ± 0.3	0.9 ± 0.2	0.9 ± 0.3
Animal protein	0.3 ± 0.2	0.3 ± 0.2	0.3 ± 0.2
Plant protein	0.6 ± 0.2	0.6 ± 0.2	0.7 ± 0.3

**Table 3:** Essential amino acid analysis.

Essential Amino Acids (g)	Average	1.2 g/kg	2.0 g/kg
Tryptophan	0.8 ± 0.3	0.7 ± 0.3	0.8 ± 0.3
Threonine	2.3 ± 0.7	2.2 ± 0.6	2.3 ± 0.9
Isoleucine	2.7 ± 0.9	2.6 ± 0.7	2.7 ± 1.0
Leucine	4.8 ± 1.6	4.7 ± 1.2	5.0 ± 1.9
Lysine	3.3 ± 1.3	3.1 ± 1.1	3.4 ± 1.6
Methionine	1.2 ± 0.4	1.1 ± 0.4	1.2 ± 0.5
Phenylalanine	3.0 ± 1.1	2.9 ± 0.8	3.0 ± 1.3
Valine	3.3 ± 1.1	3.2 ± 0.8	3.3 ± 1.3
Histidine	1.5 ± 0.5	1.5 ± 0.4	1.6 ± 0.7
Essential Amino Acids (g/kg)	Average	1.2 g/kg	2.0 g/kg
Tryptophan	0.01 ± 0	0.01 ± 0	0.01 ± 0
Threonine	0.03 ± 0	0.03 ± 0	0.03 ± 0
Isoleucine	0.04 ± 0	0.04 ± 0	0.04 ± 0
Leucine	0.07 ± 0	0.07 ± 0	0.07 ± 0
Lysine	0.05 ± 0	0.05 ± 0	0.05 ± 0
Methionine	1.2 ± 0.4	1.1 ± 0.4	1.2 ± 0.5
Phenylalanine	0.04 ± 0	0.04 ± 0	0.04 ± 0
Valine	0.05 ± 0	0.05 ± 0	0.05 ± 0
Histidine	0.02 ± 0	0.02 ± 0	0.02 ± 0

## Data Availability

De-identified data are available upon reasonable request to the corresponding author and review by the Institutional Review Board.
